# Preparation and Determination of Drug-Polymer Interaction and In-vitro Release of Mefenamic Acid Microspheres Made of CelluloseAcetate Phthalate and/or Ethylcellulose Polymers

**Published:** 2011

**Authors:** Mitra Jelvehgari, Davoud Hassanzadeh, Farhad Kiafar, Badir Delf Loveym, Sara Amiri

**Affiliations:** a *Department of Pharmaceutics, Faculty of Pharmacy, Tabriz University of Medical Sciences, Tabriz, Iran.*; b *Drug Applied Research Center, Tabriz University of Medical Sciences, Tabriz, Iran.*

## Abstract

The objective of this study was to formulate and evaluate the drug-polymer interaction of mefenamic acid (MA) using two polymers with different characteristics as ethylcellulose (EC) and/or cellulose acetate phthalate (CAP). Microspheres were prepared by the modified emulsion solvent evaporation (MESE). The effect of drug-polymer interaction was studied for each of microspheres. Important parameters in the evaluation of a microencapsulation technique are encapsulation efficiency, yield production, particle size, surface characteristics of microspheres, scanning electronic microscopy (SEM), powder X-ray diffraction analysis (XRD), and differential scanning calorimetry (DSC). The *in-vitro *release studies are performed in Tris buffer (pH 9) with Sodium lauryl sulfate (SLS). Microspheres containing CAP and EC showed 68-97% and 63-76% of entrapment efficiency, respectively. The thermogram X-ray and DSC showed stable character of MA in the microspheres and revealed an absence of drug polymer interaction. The prepared microspheres were spherical in shape and had a size range of 235-436 μm for CAP-microspheres and 358-442 μm for EC-microspheres. The results suggest that MA was successfully and efficiently encapsulated; the release rates of matrix microspheres are related to the type of polymer, only when polymers (EC and CAP combine with 1 : 1 ratio) were used to get prolonged drug release with reducing the polymers content in the microspheres. Data obtained from *in-vitro *release for microspheres and commercial capsule were fitted to various kinetic models and the high correlation was obtained in the peppas model.

Mefenamic acid, Ethylcellulose, Cellulose acetate phthalate, Microparticles, Modified emulsion-solvent evaporation.

## Introduction

Mefenamic acid (MA) is a non-steroidal anti-inflammatory drug used to treat pain, including menstrual one. It is typically prescribed for oral administration (1). MA has wide spectrum of gastrointestinal disorders (2, 3). Recently, it has been reported that MA could be used as a therapeutic agent in Alzheimer›s disease since it improves learning and memory impairment (1). Sustained release MA microspheres (4, 5), MA matrix tablets and controlled release MA-loaded alginate beads (1) have been reported in the literature. However, no commercially long-acting product exists in the market. The short biological half-life of 2 h following oral dosing necessitates frequent administration of the drug in order to maintain the desired steady state levels (2). The formulation of MA as a modified release dosage form of ethylcellulose (EC) and cellulose acetate phthalate (CAP) microspheres seems to be an alternative approach in overcoming the potential problems in the gastrointestinal tract, as it reduces the adverse effects of nonsteroidal anti-inflammatory drugs (NSAIDs) (1). Microencapsulation is a well-known method that is used to modify and delay drug release from pharmaceutical dosage forms. A great number of microencapsulation techniques are available for the formation of sustained release of microparticulate systems. One of the popular methods for the encapsulation of drugs within water-insoluble polymers is the emulsion solvent evaporation method (6). The emulsion solvent evaporation technique was fully developed at the end of the 1970s and has been used successfully in the preparation of microspheres made from several biocompatible polymers such as poly (D, L-lactide-co-glycolide) (7-11), poly (*ε*-caprolactone) (12-17) and Eudragit (18-20).

The technique of emulsion solvent evaporation offers several advantages and is preferred over other preparation methods such as spray drying, sonication and homogenization, *etc*, as it requires only mild conditions such as ambient temperature and constant stirring (6).

CAP has been widely used as an enteric coating for tablets and capsules. Lately, several workers have described investigations using CAP as a polymer employing either aqueous (21). The microencapsulation of drugs with CAP has been carried out successfully in either an aqueous or an organic vehicle. There are several methods available which may be employed in the microencapsulation with CAP and EC. They include coacervation-phase separation method, spray-drying method and extrusion method (22).

The physicochemical properties of a drug are usually the main concern in the selection of a suitable method for use. While studies evaluating drug release from microspheres prepared with individual cellulose esters have been conducted in the past (23), a comparative evaluation of drug release from microspheres prepared using a range of cellulose esters of similar molecular weights has not been available.

The purpose of this paper is MA sustained release microspheres prepared through modified emulsion solvent evaporation method (O1/O2 emulsion) and the effects of variations of drug/polymer ratio on the preparation of microspheres (using CAP/EC polymers separately and in combination to prepare different microspheres). The micromeritics properties (incorporation efficiency, yield value, particle size and distribution, surface characteristics of microspheres), powder X-ray diffraction analysis (XRD), differential scanning calorimetry (DSC) and dissolution tests were evaluated afterwards.

## Experimental

*Materials*

Mefenamic acid was obtained from Smart (Smart Pharmaceutical Company, Ningbo China), ethyl cellulose 48 cP and cellulose acetate phthalate were obtained from Sigma-Aldrich (Sigma-Aldrich, USA) and chloroform, cyclohexane, tris buffer (pH of 9), Span 80, Liquid paraffin, *n- *hexane, acetone, ethanol, orthophosphoric acid, sodium lauryl sulfate and sodium hydroxide were obtained from Merck (Merck, Germany). All solvents and reagents were of analytical grade.

*Method*

*Preparation of MA microparticles with CAP and/or EC polymers*

Microspheres were prepared through oil-in-oil (O1/O2 emulsion solvent evaporation method) using different ratios of MA to CAP and/or EC ratios (as shown in Tables 1 and 2). Liquid paraffin is preferred as an appropriate dispersing medium to ethyl alcohol and acetone, because when a solvent with a dielectric constant about 10 or above is used, non-polar liquid paraffin is preferred (24, 25). Acetone is a unique organic solvent which is polar, water-miscible and oil-immiscible. All other organic solvents like methanol, ethyl alcohol, ethyl acetate, acetone, dimethyl sulfoxide and tetrahydrofuran are oil-miscible and do not form emulsions of the polymer solution in oil (6, 9). MA was dispersed in 10 mL of the mixed solvent system consisting of acetone and ethyl alcohol in a 9 : 1 ratio (polymer solvent). The drug suspension was then emulsified in a liquid paraffin/span 80 solution under stirring at 600 rpm (Model RZR-2000; Heidolph Elektro, Kelheim, Germany) for 20 min. Then, 50 mL of chloroform or cyclohexane (non-solvent, for CAP and/or EC, respectively) was added to harden the microspheres and stirring was continued for a further 20 min. Next, the hardened microspheres were collected by filtration and washed with three portions of 30 mL of non-solvent to remove any remained oily phase, and then was air dried for 12 h.

*Determination of loading efficiency and production yield (%)*

Drug amount in microspheres was determined by dissolving 20 mg of each sample in 10 mL acetone while stirring using a mechanical stirrer at 500 rpm for 30 min. The drug concentration was determined spectrophotometrically (UV-160, Shimadzu, Japan) at 285 nm. All experiments were done in triplicate.

The loading efficiency (%) was calculated according to the following equation:

Loading efficiency (%) = (actual drug content in microparticles/theoretical drug content) × 100

The production yield of the microparticles was determined through accurately calculating the initial weight of the raw materials and the last weight of the polymeric particles obtained. All of the experiments were performed in triplicate.

*Particle size analysis*

A laser light scattering particle size analyzer (SALD-2101, Shimadzu, Japan) was used to determine the particle size of the drug and microparticle formulations. Samples were suspended in distilled water contained in a 1 cm cuvette and stirred continuously during the particle size analysis. Each sample was measured in triplicate.

*Scanning electron microscopy*

Surface morphology of microparticles was observed with a scanning electron microscope (LEO 440i, England) operating at 15 kV. The samples were mounted on a metal stub with a double adhesive tape and coated under vacuum with a platinum/palladium alloy using metallizer.

*Differential scanning calorimetry (DSC)*

DSC analysis (thermograph) of samples was done using DSC 60 instruments (Shimadzu, Japan). The samples were weighed into aluminum pans, which were closed with a pin-holed lid. Thermograms were recorded under nitrogen atmosphere from ambient to 300°C at a heating rate of 10˚C per min.

*Powder X-ray diffractometry (X-RPD)*

X-ray diffraction analysis was performed (Siemens D5000, Munich, Germany) using a nickel-filtered CuKα radiation (a voltage of 40 KV and a current of 20 mA). The scanning rate was 2°/min over a 2θ range of 20-60° and with an interval of 0.02°.

*Dissolution studies*

Dissolution was carried out using a USP basket method at 37°C and 100 rpm, in 900 mL of Tris buffer (pH 9). Microspheres (containing CAP and EC polymers separately and mixture with 1 : 1 ratio, respectively) were placed in the apparatus. Four mL of suspension was withdrawn at appropriate intervals (0. 5, 0.75, 1, 2, 3, 4, 5, 6, 7, 8 and 24 h) and each sample was returned into the apparatus. The samples were filtered through the 0.45 μm filters and used for the spectroscopic determination of the drug. Drug concentration in the samples was measured by UV spectrophotometric analysis at 285 nm. Each experiment was repeated three times.

## Results

*The effect of drug : polymer ratio on the physical properties microparticles*

Microspheres were formed after a series of steps like solvent evaporation and addition of non-solvent. Microspheres (cellulose acetate phthalate and ethylcellulose) were prepared using different drug-polymer ratios as shown in Tables 1 and 2. The drug-polymer ratio was varied through maintaining the amounts of drug, surfactant and solvent constant in all preparations and changing the amount of polymer. The results of the effect of drug-polymer ratio (microspheres containing cellulose acetate phthalate/ethylcellulose) on production yield, drug loading efficiency and mean particle size are shown in Table 3. 

**Table 1:** Mefenamic acid microsphere containing cellulose acetate phthalate formulations prepared by modified solvent evaporation method (o_1_/o_2_).

**Formulations**	**Drug : Polymer ratio**	**Emulsion (O**_1_ **/O**_2_ **)**					
		**Internal organic phase (O**_1_ **)**			**External oily phase (O**_2_ **)**		
		**Mefenamic acid (g)**	**Cellulose acetate phthalate (g)**	**Acetone (mL)**	**Ethyl alcohol (mL)**	**Liquid paraffin (mL)**	**Span 80 (%w/w)**
**F**_1_	1 : 0.75	1	0.75	9	1	200	1
**F**_2_	1 : 1	1	1	9	1	200	1
**F**_3_	1 : 1.25	1	1.25	9	1	200	1

**Table 2:** Mefenamic acid microsphere containing ethylcellulose formulations prepared by modified solvent evaporation method (o_1_/o_2_).

**Formulations**	**Drug : Polymer ratio**	**Emulsion (O**_1_ **/O**_2_ **)**					
		**Internal organic phase (O**_1_ **)**			**External oily phase (O**_2_ **)**		
		**Mefenamic acid (g)**	**Cellulose acetate phthalate (g)**	**Acetone (mL)**	**Ethyl alcohol (mL)**	**Liquid paraffin (mL)**	**Span 80 (%w/w)**
**F**_1_	1 : 0.25	1	0.25	9	1	200	3
**F**_2_	1 : 0.5	1	0.5	9	1	200	3
**F**_3_	1 : 0.75	1	0.75	9	1	200	3

**Table 3:** Effect of drug : polymer ratio on drug loading efficiency, production yield and particle size of mefenamic acid microspheres

**Formulations**	**polymer : drug ratio**	**Production yield )** **٪** **± SD)**	**Theorical drug content (** **٪** **± SD)**	**Mean amount of drug entrapped (** **٪** **± SD)**	**Drug loading efficiency (** **٪** **± SD)**	**Mean particle size **(**μm ± SD)**
**F**_1_	0.75 : 1	94 ± 2.31	57.14	39.37 ± 4.11	68.9 ± 2.02	235.58 ±1.52
**F**_2_	1 : 1	92 ± 2.68	50	36.32 ± 3.25	72.64 ± 3.52	311.76 ± 1.72
**F**_3_	1.25 : 1	90 ± 3.79	44.44	43.22 ± 4.56	97.25 ± 3.65	436.41 ± 1.34
**F'**_1_	0.25 : 1	93 ± 3.56	80	60.96 ± 2.33	76.2 ± 5.61	358.84 ± 2.31
**F'**_2_	0.5 : 1	95 ± 4.71	66.66	42.14 ± 4.52	63.21 ± 4.85	407.93 ± 4.75
**F'**_3_	0.75 : 1	98 ± 3.41	57.14	37.56 ± 5.21	65.73 ± 3.98	442.10 ± 5.94
**Mix**	0.25 : 0.25 : 1	97.23 ± 0.24	66.67	68.74 ± 9.35	103.10 ± 5.69	298.28 ± 2.35

*F_1_ to F_3_ (microspheres containing CAP), F'_1_ to F'_3_ (microspheres containing EC) and Mix (microspheres containing CAP and EC).

The pore formation is induced by diffusion of solvent from surface of the microparticles. In all of the formulations, the mean amount of drug entrapped in prepared microspheres was different from the theoretical value, since the drug loading efficiency is the range of 68.9-97.25% (microspheres containing CAP) and 63.21-76.2% (microspheres containing EC). The highest and the lowest encapsulation efficiency were obtained with acetate cellulose phthalate polymer (97.25%) and ethylcellulose polymer (63.21%), respectively. The encapsulation efficiency of the drug depended on the solubility of the drug in the solvent and continuous phase. According to Table 3, raising the polymer-drug ratio increased the production yield (when the ratio of polymer–drug increased from 0.75 : 1 to 1.25 : 1 (microspheres containing CAP) or 0.25 : 1 to 0.75 : 1 (microspheres containing EC), the production yield was 90-98% (p > 0.05)). The reason for decreased production yield at high polymer : drug ratios could be due to the decreased diffusion rate of solvents (acetone and ethyl alcohol 9 : 1) from concentrated solutions into emulsion, since through increasing the polymer amounts, the viscosity of solution was increased as well. Yield and loading efficiency of mix formulation (containing CAP and EC) were 97.23 and 103.10, respectively. The size of microspheres (containing CAP and EC) was found to be decreased by means of decreasing in the concentration of polymer CAP (Table 3). It can be attributed to the fact that with the higher diffusion rate of non-solvent to polymer solution, the smaller size of microcapsules is easily obtained (26). A volume-based size distribution of drug, polymer, and drug loaded microspheres, indicated a log-probability distribution. Mean particle size of original mefenamic acid, acetate phthalate and ethylcellulose was 145.7 ± 1.64 μm, 154.74 ± 1.56 and 125.47 ± 1.68 μm, respectively.

**Figure 1:**
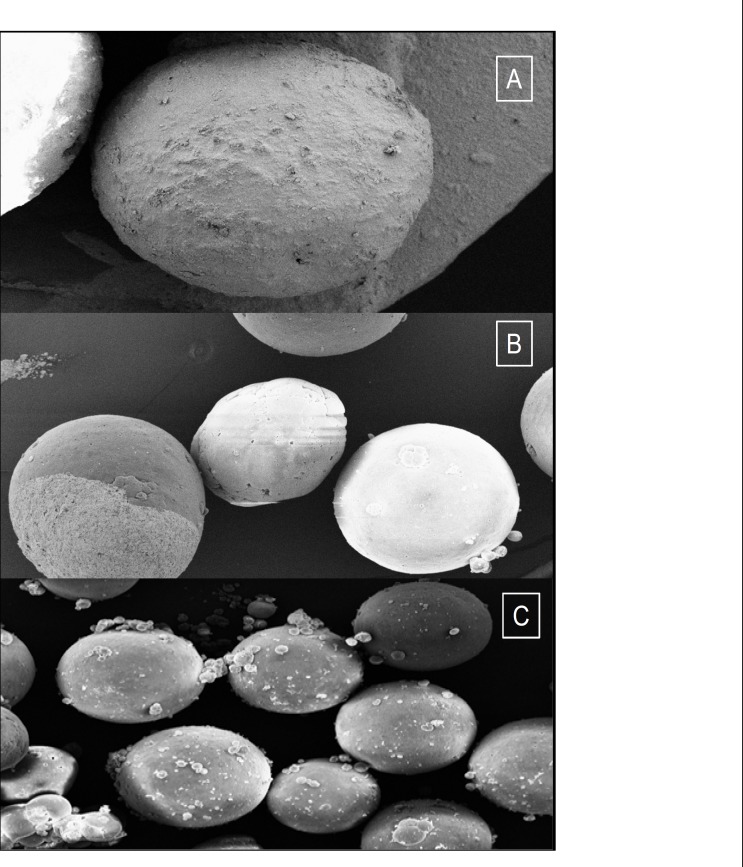
SEM of a spherical microspheres containing mefenamic acid F_1 _(polymer : drug ratio 1.25 : 1), F'_3_ (polymer : drug ratio 0.25 : 1), Mix (polymers : drug ratio 0.25 : 0.25 : 1) at 100x

SEM of microspheres (as F1, F’3 and Mix formulations) is demonstrated in Figure 1. 

When the viscosity of internal phase of these formulations was investigated, it was found that the particle sizes of microparticles were proportional with the viscosity of the dispersed phase. The results showed that the apparent viscosities of different drug : polymer ratios of microspheres containing CAP (1 : 0.75, 1 : 1, and 1 : 1.25), were 12, 21 and 32 mPa.S, respectively. The results indicated that the apparent viscosities of different drug : polymer ratios of microspheres containing EC (1 : 0.25, 1 : 0.5, and 1 : 0.75) were 15, 29 and 37 mPa.S, respectively. When the dispersed phase with higher viscosity was poured into the continuous phase (external phase), due to the higher viscosity of the internal phase, the globules of the formed emulsion might need more energy to divide into smaller particles and the bigger droplets were formed and the mean particle sizes were increased. In other studies, it was showed that the particle size depends on the solvent volume and the drug/polymer ratio, when solvent diffusion method is utilized for preparing microspheres (27, 28, 29).

*Differential scanning calorimetry (DSC)*

**Figure 2:**
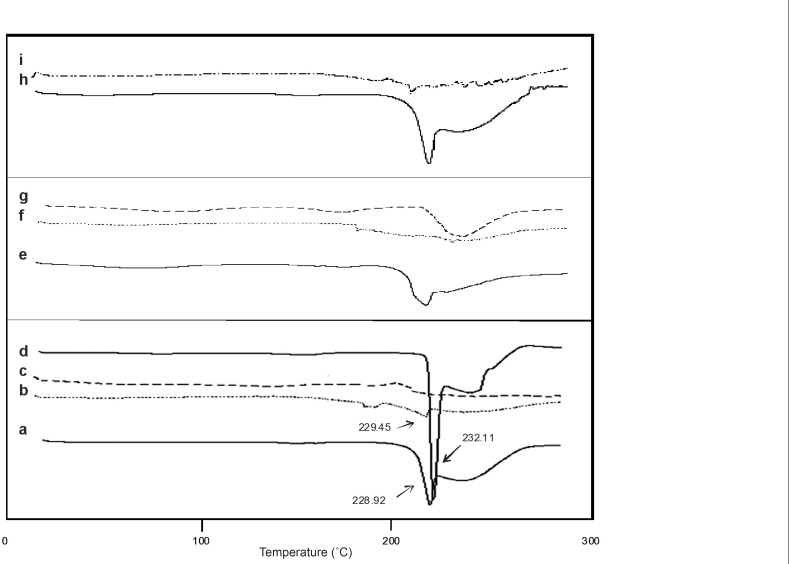
DSC thermogram of physical mixture F'_1_ (EC : MA 0.25 : 1 ratio) (a), F'_1_ (b), ethylcellulose (c), Mefenamic acid (d), physical mixture F_3_ (CAP : MA 1.25 : 1 ratio) (e), F_3_ (f), cellulose acetate phthalate (g), physical mixture (CAP : EC: 1 0.25 : 0.25 : 1 ratio) (h) and mix (i) formulations

The drug may have been dispersed in crystalline or amorphous form or dissolved in the polymeric matrix during the formation of microspheres. Any abrupt or drastic change in the thermal behavior of rather the drug or polymer may indicate a possible drug-polymer interaction (30). The endothermic peak of pure drug was observed at about 232.11°C (Figure 2). However, in the thermogram of the microparticles, (containing CAP and EC) there was an endothermic peak of the drug melting with a lower intensity than the pure drug peak, suggesting the crystalline state of the drug in the microparticles. The DSC shows the stable character of mefenamic acid in the drug loaded microspheres and revealed crystalinity form.

*X-ray powder diffractometry*

**Figure 3:**
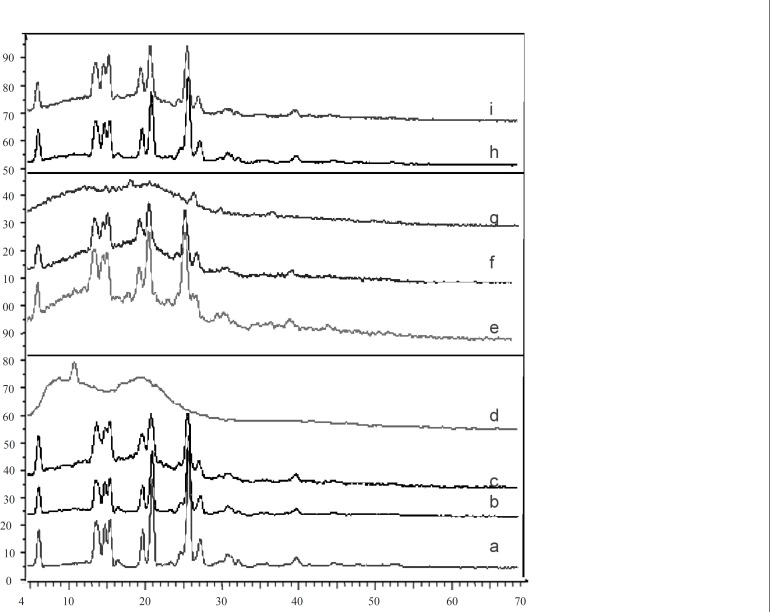
X-ray diffraction of mefenamic acid (a), F'_1 _(b), physical mixture F'_1_ (EC : MA 0.25 : 1 ratio) (c), ethylcellulose (d), physical mixture F3 (CAP : MA 1.25 : 1 ratio) (e), F_3_ (f), cellulose acetate phthalate (g), physical mixture (CAP : EC : 1 0.25 : 0.25 : 1 ratio) (h) and mix (i) formulations

The X-ray diffraction patterns of pure drug, shows that the pure drug is crystalline in nature (Figure 3). However, when it was incorporated into the polymer matrix, the principal peaks of the drug appeared with lower intensity. This could be ascribed to the crystalline state of the drug in the microparticles. It confirms the results obtained from DSC experiments.

*In-vitro release studies*

**Figure 4:**
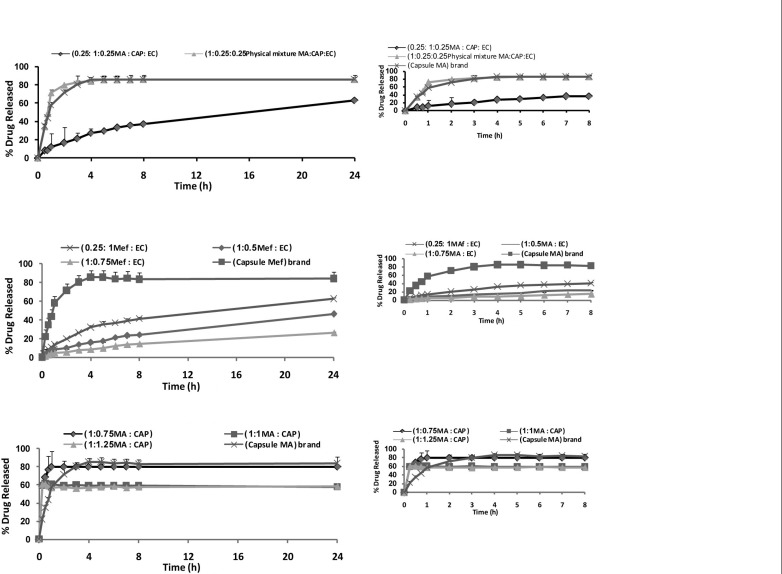
Percent release of mefenamic acid from microspheres prepared with different polymer-to-drug ratio containing cellulose acetate phthalate (A), ethylcellulose (B), combination cellulose acetate phthalate and ethylcellulose (C), physical mixture and mefenamic acid^®^ capsule

Figure 4 shows the release profile of the drug from microparticles. The *in-vitro *release of MA from microspheres containing CAP exhibited an initial burst effect, which may be due to the presence of some drug particles on the surface of the microspheres. The initial burst effect may be attributed as a desired effect to ensure the initial therapeutic plasma concentrations of drug. The release profiles are illustrated in Figure 4A. For microparticles containing EC, dissolution of MA at pH of 9 was strongly reduced, resulting in an overall slower drug release. In most cases, a biphasic dissolution profile was observed at pH of 9. The initial rapid drug leakage generally ended very early (within first 30-60 min); in the remaining time, nearly linear behavior was observed. It can be supposed that the first portion of the curves is due to MA dissolution, which starts immediately after the beginning of the test for the portion of drug on the surface of microparticles. After such a phase, two phenomena can combine in enhancing in the diffusion of the remaining dispersed drug into the bulk phase as well as the formation of pores within the matrix due to the initial drug dissolution; particle wetting and swelling which enhances the permeability of the polymer to the drug (26) (Figure 4A). The results indicated that some factors such as polymer-drug ratio governed the drug release from these microspheres. In order to keep the total surface area of the microspheres constant and thus, to get comparable results, the release studies were carried out using the same size fractions of microspheres containing equivalent amount of MA from different batches. Drug release rates increased with decreasing the amounts of MA in the formulation (containing CAP and EC polymer). Higher level of polymer corresponding to lower level of the drug in the formulation resulted in a decrease in the drug release rate (Figures 4A and 4B). As more drugs are released from the microspheres, more channels are probably produced, contributing to faster drug release rates. However, Figure 4A demonstrates that the burst effect is higher when the MA is loaded to CAP polymer. Moreover, nearly the same amount is released at 8 h from the F’3 (polymer : drug 0.75 : 1 ratio) and commercial capsule. Therefore, formulations containing CAP could not prolong the release of MA. Only formulations containing EC are prolonged release, which could be due to the thicker polymer membrane that controls the release rate (Figure 4B). One of the goals in drug microencapsulation systems development is to have an initial burst release and achieve a constant release rate thereafter. The degree of initial burst fro m the microparticles depends on the drug encapsulate ability of the polymer matrix, which thereby, making it unavailable for immediate diffusion (29). For this reason, efforts to reduce the initial burst have followed in the same track as those, increasing encapsulation efficiency, so, understanding the previous effort to maximize the encapsulation efficiency will thus be useful in controlling the release profile. Combination of CAP and EC corresponding to the lower level of the polymer with MA in the formulation (Mix) resulted in the sustained release and reduced the initial release (Figure 4C). Statistical analysis of data was performed by comparing the dissolution efficiency (DE), dissolution time for 50% fractions of drug (t_50%_), and “similarity factor, f2 (used to compare multipoint dissolution profiles)” (Table 4) (31). DE was calculated from the area under the dissolution curve at time and expressed as the percentage of the described rectangle area by 100% dissolution in the same time. F1 and F›3 microspheres showed a lower dissolution efficiency 40.89 and 24.25%, respectively and a slow dissolution. MA capsule ® and physical mixture had a higher release in comparison with microspheres containing CAP and/or EC (p < 0.05), (Table 4 and Figure 4). Physical mixtures of MA containing EC and combination EC and CAP were similar with MA capsule ®.

**Table 4:** Comparison of various release characteristics of mefenamic acid from different microsphere formulations, physical mixture and mefenamic acid ^®^ capsule

**Formulation**	**T** ^ b^_ 50% _ **(h)**	**DE** ^c^ ** (%)**	**Q** ^d^_0.5_ **(%)**	**Q** ^e^_s_ **(%)**	**Similarity factor**
**F**_1_	0.5	55.98 ± 2.41	68.82 ± 0.58	80.11 ± 1.09	40.85
**F**_2_	0.5	40.89 ± 6.52	61.30 ± 1.99	62.50 ± 1.17	40.89
**F**_3_	0.5	41.79 ± 5.24	60.71 ± 1.06	60.88 ± 2.61	33.72
**PMa (CAP)**	0.5	87.45 ± 6.91	46.71 ± 8.44	87.62 ± 5.52	47.5
**F'**_1_	24	3.69±55.02	8.82 ± 0.58	41.04 ± 2.16	20.3
**F'**_2_	> 48	40.92 ± 5.27	4.44 ± 0.12	24.22 ± 0.05	14.85
**F'**_3_	24	24.25 ± 4.19	2.63 ± 0.36	14.39 ± 0.41	11.77
**PM (EC)**	2	79.05 ± 2.33	23.69 ± 1.74	81.49±1.51	54.24
**Mix**	8	54.34 ± 1.54	7.97 ± 0.07	37.18±1.01	18.77
**PM (CAP and EC)**	0.75	84.12 ± 2.17	33.93 ± 2.59	85.95±6.21	63.17
**Capsule MA** ^®^	1	84.12±5.64	33.90±3.57	85.68±6.22	100

^a^PM: Physical Mixture; ^b^t_0.5%_: dissolution time for 50% fractions ^c^DE: Dissolution Efficiency ^d^Q_0.5_: amount of drug release after 2h; ^e^Q_8_: amount of drug release after 8h.

The *in-vitro *release profiles were fitted on various kinetic models in order to find out the mechanism of drug release (32, 33). The fit parameters of Higuchi, first-order, Peppas and zero-order equations are given in Table 5. The rate constants were calculated from the slope of the respective plots. A high correlation was observed for the Peppas model. The obtained data were also put in Korsemeyer-Peppas model in order to find out n-value, which described the drug release mechanism. The n-value of microspheres of different drug to polymer ratio and conventional capsule was between 0.53 and 0.61 which indicates that the mechanism of the drug release was diffusion and erosion controlled.

**Table 5:** Fitting parameters of the *in-vitro *release data to various release kinetic models from different microsphere formulations, physical mixture and mefenamic acid ^®^ capsule

**Order**		**F**_1_	**F**_2_	**F**_3_	**F'**_1_	**F'**_2_	**F'**_3_	**mix**	**MA** **Capsule**
**Zero** **f = kt**	K	0.0001	0.0001	0.0002	0.0116	0.0071	0.0121	0.0131	0.0056
	RSQ	0.9134	0.9289	0.7380	0.9256	0.9397	0.7589	0.8108	0.1742
	٪D(SS)	870.5522	842.2092	1002.43	794.2498	767.7882	924.2972	877.9097	1127.4706
**First** **ln(1-f) = kt**	K	0.0003	0.0001	0.0003	0.01840	0.0091	0.0231	0.0248	0.0196
	RSQ	0.9692	0.9585	0.8682	0.9741	0.9654	0.8798	0.9099	0.2101
	٪D(SS)	709.6815	759.0672	846.7337	936.9552	686.7225	770.4423	701.1172	998.4947
**Peppas lnf = lnk + blnt**	b	0.5853	0.6116	0.5676	0.5455	0.5680	0.5269	0.5689	0.7225
	K	0.0064	0.0031	0.0126	0.0768	0.0424	0.1280	0.1143	0.5625
	RSQ	0.9762	0.9786	0.9785	0.9830	0.9891	0.9828	0.9883	0.9731
	٪D(SS)	119.3664	127.6362	96.3153	87.6224	78.3164	66.1235	60.4413	9.6819
**Higuchi f = kt** ^0.5^	K	0.0118	0.0072	0.0136	0.0916	0.0562	0.1022	0.1088	0.0614
	RSQ	0.9941	0.9957	0.9303	0.9936	0.9953	0.9313	0.9575	0.3631
	٪D(SS)	254.4600	386.8386	295.0244	157.3727	257.2416	308.6438	160.8636	967.2176

*F_1_ to F_3_ (microspheres containing CAP), F›_1_ to F›_3_ (microspheres containing EC) and Mix (microspheres containing CAP and EC).

## Conclusion

MA microspheres were prepared using the modified solvent evaporation method. MA microspheres (containing combination of CAP and EC) could be prepared with high drug encapsulation efficiency. Polymer : drug ratio influenced the sphericity of the microspheres. The yield and entrapment efficiency were high for Mix formulation (containing CAP and EC). It was observed that increasing the polymer concentration leads to an increase in the mean particle size of the microspheres. Among these microspheres, the EC (F›1) and the EC and CAP (Mix) microspheres exhibited a similar sustained release effect of the commercial product via *in-vitro *dissolution. Therefore, the optimal release profile might be obtained by the combination of CAP and EC microspheres. The drug release from CAP and EC microspheres exhibited a lower initial burst effect and the mechanisms of the drug release – diffusion and erosion – were controlled. The controlled release without the initial peak level that is achieved with these formulations may reduce dose frequency and side effects as well as improving the patient’s compliance.

## References

[1] Sevgi F, Yurdaiper A, Kaynarsoy B, Turunç E, Güneri T, Yalçin A (2009). Studies on mefenamic acid microparticles: formulation, in-vitro release, and in-situ studies in rats. AAPS Pharm. Sci. Tech.

[2] Reynolds JEF (1998). Martindale: the Extra Pharmacopeia.

[3] Jelen MG, Jaming D, Schabus H, Pipam W, Likar R (2008). A comparison of the efficacy and rate of side-effects of mefenamic acid and naproxen in adult patients following elective tonsillectomy: a randomized double-blind study. Acute Pain.

[4] Lalla JK, Ahuja PL (1991). Drug targeting using non-magnetic and magnetic albumin-globulin mix microspheres of mefenamic acid. J. Microencapsul.

[5] Sevgi F, Ozyazici M, Kaynarsoy B, Ozyurt D, Pekçetin C, Salman Agba D, Hounslow Mike J, Seville Jonathan PK (2006). Histological Evaluation of Drug-Loaded Alginate Beads and Eudragit Microspheres. Proceedings of the 13th International Pharmaceutical Technology Symposium; Antalya, Turkey (2006) 10-13 Sep.

[6] Kim BK, Hwang SJ, Park JB, Park HJ (2002). Preparation and characterization of drug-loaded polymethacrylate microspheres by an emulsion solvent evaporation method. J. Microencapsul.

[7] Herrmann J, Bodmeier R (1998). Biodegradable, somatostatin acetate containing microspheres prepared by various aqueous and non-aqueous solvent evaporation methods. Eur. J. Pharm. Biopharm.

[8] Kawashima Y, Yamamoto H, Takeuchi H, Hino T, Niwa T (1998). Properties of a peptide containg dl-lactide/glycolide copolymer nanospheres prepared by novel emulsion solvent diffusion methods. Eur. J. Pharm. Biopharm.

[9] Viswanathan NB, Thomas PA, Pandit JK, Kulkarni MG, Mashelkar RA (1999). Preparation of non-porous microspheres with high entrapment efficiency of proteins by a (water-in-oil)-in-oil emulsion technique. J. Control. Rel.

[10] Murakami H, Kobayashi M, Takeuchi H, Kawashima H (2000). Further application of a modified apontaneous emulsification solvent diffusion method to various types of PLGA and PLA polymers for preparation of nanoparticles. Powder Tech.

[11] Yang YY, Chia HH, Chung TS (2000). Effect of preparation temperature on the characteristics and release profiles of PLGA microspheres containing protein fabricated by double emulsion solvent extraction/evaporation method. J. Control. Rel.

[12] Benoit MA, Baras B, Gillard J (1999). Preparation and characterization of protein-loaded poly(ε-caprolactone) microparticles for oral vaccine delivery. Int. J. Pharm.

[13] Chen DR, Bei JZ, Wang SG (1999). Polycaprolactone microparticles and their biodegradation. Poly. Degrad. Stab.

[14] Lamprecht A, Ubrich N, Pérez MH, Leher CM, Homan M, Maincent P (2000). Influence of process parameteters on nanoparticles preparation performed by a double emulsion pressure homogenization technique. Int. J. Pharm.

[15] Pérez et MH, Zinutti C, Lamprecht A, Ubrich N, Homan A, Bodmeier R, Maincent P (2000). The preparation and evaluation of poly(ε-caprolactone) microparticles containing both a lipophilic and hydrophobic drug. J. Control. Rel.

[16] Iooss P, Ray AML, Grimandi G, Daculsi G, Merle C (2001). A new injectable bone substitute combing poly(ε-caprolactone) microparticles with biphasic calcium phosphate granules. Biomaterial.

[17] Youan BBC, Jacson TL, Dickens L, Hernandez C, Ababio GO (2001). Protein release profiles and morphology of biogradable microcapsules containing an oily core. J. Control. Rel.

[18] Das SK, Das NG (1998). Preparation and in-vitro dissolution profile of dual polymer (Eudragit RS 100 and RL 100) microparticles of diltiazae hydrochloride. J. Microencapsul.

[19] Lamosa MLL, Lόpez CR, Jato JLV, Alonso MJ (1998). Design of microencapsulated chitosan microspheres for colonic drug delivery. J. Control. Rel.

[20] Lee JH, Park TG, Choi HK (2000). Effect of formulation and processing variables on the characteristics of microspheres for water soluble drugs prepared by w/o/o double emulsion solvent diffusion method. Int. J. Pharm.

[21] Joseph W, Nairn JG (1986). Some factors affecting the microencasulation of pharmaceuticals with cellulose acetate phthalate. J. Pharm. Sci.

[22] Wan LSC, Chui WK (1995). Deviation of the ratio of drugs in a two-component mixture encapsulated in cellulose phthalate microspheres. J. Microencapsul.

[23] Bhardwaj SB, Shukla AJ, Collins CC (1995). Effect of varing drug loading on particle size distribution and drug release kinetics of verapamil hydrochloride microspheres prepared with cellulose esters. J. Microencapsul.

[24] Bogataj M, Mrhar A, Krist A, Kozjek F (1991). Eudragit E microspheres congaing bacampicillin: preparation by solvent removal methods. J. Microencapsul.

[25] Kim CK, Kim MJ, Oh KH (1994). Preparation and evaluation of sustained release microspheres of terbutaline sulfate. Int. J. Pharm.

[26] Pignatello R, Consoli P, Puglisi G (2000). In-vitro release kinetics of Tolmetin from tabletted Eudragit microparticles. J. Microencapsul.

[27] Barkai A, Pathak YV, Benita S (1990). Polyacrylate (Eudragit Retard) microspheres for oral controlled release of I. Formulation desgin and process optimizationnifedipine. Drug Dev. Ind. Pharm.

[28] Pongpaibal Y, Price JC, Whitworth CW (1984). Preparation and evaluation of controlled release indomethacine microspheres. Drug Dev. Ind. Pharm.

[29] Bijanzadeh M, Mahmoudian M, Zolfaghari ME, Gouya MM, Khazinia T, Khosravi A (199). The Influence of Particle Size and Dissolution Rate on Bioavailabilty of Two Indomethacine Capsules. DARU.

[30] Yeo Y, Park K (2004). Control of encapsulation efficiency and initial burst in polymeric microparticle systems. Arch. Pharm. Res.

[31] Moore JW, Flanner HH (1996). Mathematical comparison of dissolution profiles. Pharm. Technol.

[32] Yuksel N, Kanik AE, Baykara T (2000). Comparison of in-vitro dissolution profiles by ANOVA-based, model dependent and independent methods. Int. J. Pharm.

